# Women’s experiences on the use of Implanon as a contraceptive method in a selected primary healthcare facility in KwaZulu-Natal

**DOI:** 10.4102/curationis.v44i1.2187

**Published:** 2021-11-08

**Authors:** Lucky N. Mgobhozi, Pretty N. Mbeje, Gugu G. Mchunu

**Affiliations:** 1 School of Nursing, Faculty of Health Sciences, Walter Sisulu University, Umthatha, South Africa; 2 School of Nursing and Public Health, Faculty of Health Sciences, University of KwaZulu-Natal, Durban, South Africa; 3 Faculty of Health Sciences, Durban University of Technology, Durban, South Africa

**Keywords:** Implanon, Implanon users, experiences, perceptions, primary healthcare

## Abstract

**Background:**

The South African department of health recently introduced subdermal Implanon contraceptive implant with the aim to reduce teenage pregnancy and maternal mortality. First used in all public healthcare facilities across the country since early 2014, this method of contraception has been described as highly effective. However, some women have reported unbearable side effects, forcing them to remove the contraceptive implant early before its expiry date. Negligible emphasis has been placed on staff training and development to equip the nurses with new protocol and policies on Implanon.

**Objectives:**

The objective of this study was to explore experiences of women using Implanon as method of contraception at a selected primary healthcare facility in KwaZulu-Natal province of South Africa.

**Methods:**

A qualitative, descriptive and exploratory study design was used. A purposive sampling technique was used and a sample of seven women aged between 15 and 50 years was selected for this study. Semi-structured interviews were used in the data collection process. The Tesch’s method for data coding and data analysis was utilised. Necessary ethical measures were taken to ensure that the study is trustworthy. The study was conducted at Community Health Centre, KwaZulu-Natal between June 2017 and December 2018.

**Results:**

The findings showed that some participants were still willing to continue using this method of contraception regardless of the unwanted side effects. Major side effects reported were heavy menstrual bleeding, pain and discomfort, weight loss, insomnia and decreased sexual interest, which resulted in most participants stopping the use of Implanon.

**Conclusion:**

Most of the participants’ experience unwanted side effects because of poor screening, counselling and support. There is a clear demand to develop a screening tool and facilitate training of healthcare workers when initiating the use of Implanon.

## Background of the study

Every day, about 800 women die because of pregnancy and childbirth-related complications, with almost 99% of these cases occurring in developing countries (Alemayehu et al. 2015; Haddou 2014; WHO 2014). An estimated 200 million women in developing countries use contraceptive methods, but there are still barriers to access such services. Some of these barriers are transport challenges for most women who live in remote areas (UNFPA 2015). Kartz (2015) argued that in some instances, supply chains cut off remote rural areas where the poorest segment of low-income population resides. The prohibitive cost of contraceptives in many developing countries is a prominent contraceptive access barrier to women (Kartz 2015). The above-mentioned barriers mainly affect women who come from disadvantaged communities (Abiodum & Balogum 2009; Imo, Isiugo-Abanihe & Onabanjo 2013).

It has been argued that a large number of maternal deaths could be averted by following family planning, particularly long acting contraceptive methods. The efficacy of these methods lie in their ability to reduce visits to healthcare facilities. They are also highly effective and cheap. Implanon is one of the many such contraceptives that have been introduced. The contraceptive has been available since 2014 (South Africa; South African Department of Health 2015). The total uptake of Implanon estimated by the Department of Health for 2018 was 433 000 women, but the total demand was as high as 567 000 (Chola et al. 2019). However, such statistics indicate that women are in great need of long-lasting contraceptive method. Despite this high percentage of users, an increasing number of women have recently reported unwanted side effects in the past subsequent years it was introduced and decrease of almost 50% of insertion numbers after a year (Pleaner et al. 2017).

Implanon is a birth control contraceptive implant – small flexible plastic rod, the size of a matchstick – inserted subdermally under the skin of the upper arm that releases small amounts of progestogen into the body for a period of three years (Patel 2014). It inhibits ovulation in the ovaries, thus causing changes to the cervical mucus so that spermatozoa does not reach the uterine cavity. Implanon consists of a single ethylene vinyl acetate copolymer rod that is 2 mm in diameter and 4 cm long (Gbolade 2010; Patel 2014; Spies et al. 2010).

Patni et al. (2006) argued that Implanon’s failure result from potent-enzyme inducers known to have deleterious effects leading to intrauterine or ectopic pregnancy. However, two of these pregnancies were directly related to a concomitant use of rifampicin. Recent studies show that other drugs that have contributed to the failure of Implanon include: anti-epileptic drugs (phenytoin, phenobarbital, primidone), antibiotics (rifampicin, rifabutin), antifungal drugs (griseofulvin), protease inhibitors (lopinavir, atazanavir, amprenavir) and non-nucleoside reverse transcriptase inhibitors (efavirenz, nevirapine). A research case report on the failure of Implanon on anti-tuberculous therapy by Gbolade (2010) established that drug interaction with hormonal contraceptives are challenging when steroid metabolism is stimulated. However, in the liver enzyme system cytochrome P450 plays a significant role in drug metabolism, and drugs that induce these enzymes cause increased elimination of contraceptive steroids, which adversely lead to reduced reliability and unplanned pregnancy. There are several factors that counteract effectiveness of Implanon in the body such as certain drugs used to treat epilepsy, tuberculosis and antiretroviral drugs, as also poor screening and lifestyle factors (alcohol consumption and sport).

Mukuka (2015) argued that some women in South Africa have experienced side effects of the Implanon device and are not convinced of this new contraceptive implant in terms of effectiveness. However, clinical evidence indicates that it is common for women to experience side effects for the first three months after insertion of this implant (Patel 2014). But there are still high numbers of women who experience side effects because of Implanon, forcing them to remove the implant before its expiry (Patel 2014; Sikosana 2015; UNFPA 2015). Another investigation by Sikosana (2015) revealed that some clinics have stopped providing Implanon contraceptive implant because there were many issues raised regarding its use, and some issues are still unclear between the healthcare providers and clients. Another challenge is that there has been little emphasis placed on staff training and development to equip the nurses with new protocol and policies on Implanon. The above-mentioned argument clearly supports the need for this study to be conducted because this is a fairly new contraceptive method and there seems to be gaps in the service provision.

Patel (2014) conducted a study on contraceptives and personal responsibility in South Africa. It was revealed that the main issue with the Implanon implant is the change in bleeding patterns and this adverse effect is responsible for women discontinuing its use, especially if they were not adequately counselled. Whilst no pregnancies were reported in the study population, pregnancies reported were related to insertion issues. Implanon was found to be safe for use in obese and hypertensive women. Similarly, in a study conducted on experiences with Implanon in Southern Nigeria (Ojule, Oranu & Enyindah 2012) it was found that women who are currently on Implanon experienced unwanted side effects, such as vaginal spotting 60%, menorrhagia 13.3% and inter-menstrual bleeding 13.3%. Amongst the participants 51.8% of the clients changed from other contraceptive methods to Implanon. Other participants discontinued Implanon during the study period, whereas the continuation rate was 94.6% (participants who accepted Implanon). This also indicates client satisfaction, acceptance and continuation with Implanon.

### Aim and objectives

This article aimed to explore describes the experiences and perceptions of women using Implanon at a selected primary healthcare facility in KwaZulu-Natal, South Africa.

### Method

A triangulation of convergence model was used where the researchers collected and analysed quantitative and qualitative data separately. The scope of this article is on the qualitative aspect of the study, which explored the experiences of the users.

The exploratory descriptive design using qualitative approach was employed to analyse qualitative data collection. This method was used to obtain experiences of women using Implanon contraceptive method.

### Study participants and setting

The target population were all clients at the clinic who had inserted Implanon in the past 6 weeks, have been visiting the clinic for follow up, and individuals who had removed Implanon as a result of other problems.

Participants were sampled purposively based on their first-hand experience with Implanon use. Selected participants were clients who were being served by the clinic at the time of data collection. The sample size was determined by the saturation of the data, and data saturation was reached in the 12th day of data collection. Four participants were pretested but the results were not used in the main study; seven participants participated in this study.

The setting for the study was a community healthcare facility located in uMgungundlovu district of KwaZulu-Natal. Most of the clients who are seen at this community healthcare centre belong to the middle-income class and the majority is well educated (South African Department of Health 2014). However, this community healthcare centre was chosen because it has higher uptake of Implanon contraceptive and is located centrally in town. The clients who use these community healthcare centres generally come for minor and alignment consultation, integrated management of childhood illness, family planning, chronic illness, as well as other primary healthcare services. The data collection was performed in the family planning department focusing on Implanon users, who were coming for follow up consultation, and any other clients readily available at the clinic meeting the recruitment criteria.

### Data collection

The data collection process took place after obtaining ethical approval and permission from the relevant authorities to conduct the study. On obtaining the ethical clearance, the researcher approached the management of the selected clinic situated at uMgungundlovu District to arrange for data collection process commencement. Data collection took place at a selected community healthcare centre in a Family Planning department. The data collection venue where interviews were conducted was also identified and suitable interview dates were confirmed. Data collection process was conducted over a period of 12 days until the data saturation was reached. Sample size was not pre-determined.

The researcher explained the purpose and the process of the data collection to the staff allocated at the family planning clinic on the day of the data collection. The researcher waited upon each client coming in for family planning services in the waiting area; specifically, those coming for consultation or review were asked to participate in this study. Then those women with Implanon were asked to follow the researcher into a consultation room, where data collection procedure took place. In the consultation room, everyone was provided with a chair and a table to sit at. Thereafter, all the women who met the recruitment criteria were briefed on the study and asked if they were willing to participate. Those who were willing were then asked to sign consent forms before they participated in the study.

An interview guide was used to obtain information from the willing participants for approximately 15–20 min. The interview guide contained semi-structured questions, with the researcher probing where necessary to obtain more clarity and in-depth information (Brink, Van der Walt & Van der Rensburg 2012). The main interview question for this study was ‘What is your experience with the use of Implanon contraceptive?’. Other questions were just probes seeking clarity from the participants. The pilot study of the interview guide was performed by researcher with four participants at the clinic before data collection. This was carried out to determine whether questions and directions were clear to the participants. There was no relationship between the researcher and the participants. Therefore, selection of participants was fair and free of offence or threats.

### Pilot study

In this study, a pilot study was carried out with four participants prior to data collection. The results of the pilot study were not included in the main study. The pilot study showed the suitability of the instrument positively with no difficulties imposed on the participants. Participants were clear with the questions and probing or questions seeking clarity were asked.

## Trustworthiness

Trustworthiness was ensured by following a certain criterion: credibility, transferability, dependability and confirmability (Brink et al. 2012; Polit & Beck 2008).

### Credibility

To ensure credibility, the researcher made notes and recorded interviews using audiotapes to make sure that information was thorough and correct. Interviewer was making notes during each interview and used audiotape to record the interviews after obtaining consent from the participants. Participants confirmed the information after transcription. This was done to ensure that the themes generated from the information obtained accurately represented the views of the participants.

### Transferability

The researcher ensured the trustworthiness of the findings by sharing the findings with all healthcare practitioners who did not participate in the study for constructive criticism.

### Dependability

Data collection was performed on participants who had used Implanon for more than 6 weeks. All participants were able to share their experiences because they understood Implanon contraceptive. An audit trail was maintained by ensuring that raw information that was collected from each participant was kept safe for future reference.

### Confirmability

Confirmability was based on the characteristics of data being dependable. Raw data were confirmed by the use of direct quotes from the raw data to eliminate subjectivity. Co-coder was utilised in the analysis.

## Data analysis

Tesch’s method of eight steps in data analysis was adopted. In developing themes, the researcher was guided by the theoretical framework and research objectives. The following were investigated in order to answer the research questions:

- Experiences of women using Implanon
- Perceived barriers to the use of Implanon
- Perceived enablers to the use of Implanon

The following covers the theoretical framework guiding the study:

- Cues of action
- Efficacy

The following section details the process of how data analysis was undertaken in this study.

Data were analysed according to Tesch’s eight steps of data analysis whereby the researcher carefully read through all the transcriptions, making notes of ideas that came to mind. The researcher selected one interview and read it to try to extract meaning from the information, writing down thoughts that would come to mind. Similar topics were arranged in groups, forming columns labelled: major topics, unique topics, and leftovers, respectively. The researcher found the most descriptive wording for the topics and converted them into categories.

There are five different stages, namely (1) familiarisation with the material, (2) formulation of emergent themes, (3) coding of different themes, (4) charting, cutting, pasting and rearrangement of data under different themes and (5) interpretation and explanation of findings.

### Results

Experiences were either positive (enablers) or negative (barriers). The participant’s responses were categorised and sub-categorised into themes which are detailed as follows.

### Participants’ demographics

In this study, four participants were used for pilot study and seven participants were interviewed for the actual study. The age range was between 15 and 50 years. There were three married participants whilst the rest were not married. Four of the participants were unemployed whilst the other three were employed. Five of the participants were Christian, one was Muslim and one belonged to the Zulu traditional religion. All the participants were women using Implanon contraceptive for family planning.

### Perceived enabling experiences

Most participants were satisfied with using Implanon, others even complemented the positive staff attitude and assistance by nurses. Of note was that these participants reported unwanted side effects but nevertheless, they were happy with the method as they showed positivity (all citations hereafter are verbatim):

‘I only came to the clinic because of side effects. Besides that, I was happy with having the implants on my body.’ (34 years, single, female)

‘I had a very good experience with Implanon except during [*the*] menstrual period. I still want to use it even now.’ (46 years, married, female)

‘I can advise others to use the implant. It worked very well and I had a good experience with it.’ (17 years, single, female)

### Positive staff attitude

Despite the side effects, positive staff attitude surpassed it all. Participants were extremely pleased with nurse’s attitude and assistance when they visited the healthcare clinics for help:

‘Nurses were very helpful to me, they explained everything before Implanon was inserted, and when I returned, they were willing to help. I am happy.’ (22 years, single, female)

‘Staff attitude I saw in everyone today left me satisfied with Implanon.’ (29 years, married, female)

### Experiences perceived as barriers

Some participants were reluctant to use this method of contraception because of the side effects. Some felt that Implanon worked well with other people whilst they were the only ones experiencing severe side effects. As such, the method was viewed as working differently on different people. These participants shared their negative experiences in relation to menstrual bleeding/periods:

‘I was on menstrual periods continuously because of this new injection on my arm … and my weight dropped significantly since then.’ (50 years, married, female)

‘In a day I changed maybe four to five sanitary pads when I had heavy menstrual bleeding …’ (25 years, single, female)

### Ineffective treatment of symptoms

Participants indicated that they were forced to return to the clinic with the same problem of intermittent menstrual bleeding but there was no effective treatment as it usually stopped for a while after taking treatment from the clinic:

‘I had continuous menstrual bleeding for the past 4 months, then last month it stopped after taking tablets from the clinic but it started again.’ (50 years, married, female)

‘I was up and down to the clinic but they couldn’t help me … I was given contraceptive pills, they told me to drink yellow pills not red ones which I had to take once or twice a day.’ (25 years, Single, female)

‘At another clinic they said discharge has nothing to do with the implant. But where I inserted the implant they said it’s common to have a discharge or headache.’ (34 years, single, female)

### Unexplained weight loss

Some participants were very concerned about the unplanned weight loss after starting this method of contraception. This was viewed as a reason to remove the device:

‘… when I came to the clinic another male nurse checked my weight and he observed that it had dropped too much and he advised me to have it removed at the hospital …’ (46 years, married, female)

‘It was very painful and sad to be bleeding and losing weight every time, yet when you seek help you don’t get it …’ (22 years, single, female)

### Discomfort and pains

Some participants reported that they experienced headaches and body pains, which they believed were because of the use of the implant i:

‘… I had headache, loss of weight, pains on my shoulders after having continuous menstrual bleeding for about a month.’ (34 years, single, female)

‘I have been experiencing dizziness, loss of appetite, lower abdominal pain, acne, headache and sweating. Lastly, continuous menstrual periods. But they only gave me tablets for heavy bleeding and headache.’ (17 years, single, female)

### Neurological symptoms

Whilst these symptoms were not directly linked to the method of contraception, participants believed these symptoms were effects of Implanon because it was confirmed by other users:

#### Memory loss

Some participants complained about memory loss and stated that:

‘… this injection can make you forget things easily, you just become insane, for I was forgetting where I kept my money most of the time.’ (29 years, married, female)

‘… I wasn’t aware until another girl from my church told me that she was forgetting the names of her colleagues at work. I have heard many reporting the same problem that the injection causes you to forget things whilst it is inside you.’ (25 years single, female)

#### Insomnia

Some participants complained that they could not sleep at night. This happened when they were experiencing other side effects and it happened without any clear reference as a cause:

‘… at night I couldn’t sleep at all.’ (46 years, married, female)

‘I also had loss of weight, memory loss; sweating and I couldn’t sleep well.’ (29 years, married, female)

### Effects on sex life

Participants also reported that they wanted no sexual contact whilst others reported lack of sexual desire with their partners after they have had the implant inserted on them. In most cases, they wanted no sexual intimacy:

‘… I had no desire for sex at all ever since …’ (34 years, single, female)

‘Most of the times I had no desire to have sex; it also started when I was using the implant.’ (17 years, single, female)

### Mismanagement of side effects

For some participants the experience of visiting more than one health facility because of their side effects was more discouraging as they would get the same treatment over and over again without any relief of symptoms:

‘I decided to visit the clinic regarding this menstrual period problem, and I was told it will go away after time. It’s just a side effect.’ (34 years, single, female)

‘When I had this problem, I went to the other clinic where they told me they couldn’t take it out. They said I should go to Eastern Cape where I got the implant. I went back, some other day and another nurse gave me contraceptive tablets to stop vaginal bleeding … they told me to go to another clinic if I want to remove it.’ (50 years, married, female)

### Severity of side effects

According to some participants, the side effects were severe enough to get the device removed. However, the nurses at the clinics were not willing to remove it:

‘I came back to the clinic to remove the implant and then they refused to remove it. They told me about the procedure they have to follow. They gave me tablets. I went back again then they told me I had quickly returned as I should have allowed treatment to work first until they remove it after 3 years.’ (22 years, single, female)

‘I tried hard to get the implant removed, every clinic referring me to another. I learnt a lesson. I decided to remain patient until a solution was found.’ (29 years, married, female)

### Staff attitude

The nurses’ attitude when participants asked for help with removal of the device was a concern as healthcare workers either refused to remove the device or blamed them for wasting government’s resources:

‘Nurses told me that I was wasting government money for this implant. They said it was very expensive. They also said if I go to private it’s too expensive. Why are you taking advantage over short period you have had it.’ (17 years, single, female)

‘… they book three per day for removal but whenever we come for bookings we get very distant dates.’ (22 years, single, female)

### Lack of support

Implanon users revealed that they were not educated or counselled about the implant, they therefore ended up wanting to remove the Implanon too soon before its expiry. This is how they responded:

‘I had an abortion in 2014, when a nurse called us all those who were there to use new implant. She didn’t explain to us how it worked and did not give us other information we were supposed to know.’ (22 years, single, female)

‘Another thing happened, I got it very late that only HIV negative women are free to insert Implanon but it was not explained initially to me.’ (50 years, married, female)

‘If it was wrong for me to use antibiotics and other medication they should have told me before because I didn’t know whether to take medicine or not.’ (34 years, single, female)

### Misconceptions and rumours from clients

It was found that various rumours and misconceptions were spread amongst the women using the implant. Some participants were told by their peers that it is possible to get pregnant whilst using the implant. This is how they responded:

‘My friend told me that it is possible to be pregnant whilst using the implant because there were people she knew who fell pregnant whilst using the implant. She said it is possible to happen, really …’ (34 years, single, female)

‘Yesterday, another friend of mine told me that it is possible to get pregnant whilst using the implant. Then, I asked her, how come I am not pregnant, because I am also using the implant. She responded that it differs from one person to another.’ (25 years, single, female)

### Parter disapproval and violence against women

Amongst the participants it was reported that some women experienced some violence and disapproval for using Implanon. It was found that women were forced to remove the implant and razors or needles were used whilst trying to remove the implant. This is how they responded:

‘My husband was fine when I first told him that I am using the implant, suddenly his mind changed and told me that he won’t continue with lobola negotiations until I remove the implant. I then promised him to go to the clinic.’ (25 years, single, female)

‘My partner didn’t want me to use the implant. He tried to remove it by using a razor to cut a small opening and he then used a needle to remove the implant. When the blood was coming out, I told him to stop because it might be dangerous and risky to remove it. I was so disappointed when I got a very far booking in August.’ (34 years, single, female)

### Dissatisfaction with Implanon

Most participants who were interviewed in this study reported they were dissatisfied with Implanon. The common reasons for their dissatisfaction were: side effects, failure and other potential risk suspected by users. This is how they responded:

‘I won’t use it again; I don’t think it will be good for me … I am not satisfied at all with the implant because of the continuous menstrual bleeding. I won’t use it again; I don’t think it will be good for me.’ (22 years, single, female)

‘It works differently with people. Some are not happy, others are happy, just like myself. It is a matter of choice; a person must choose what works for her.’ (29 years, married, female)

‘At the beginning of the year 2016, I felt so sick at all times. I experienced fever, cough, vomiting after eating and dizziness. So I came to the clinic with the problem then the nurse at the clinic suggested for urine test. I was then checked and I found out that I was pregnant. I was surprised because I was using the implant. I will never use Implanon, because I got pregnant. Although I was told that I won’t get pregnant for the period of 3 years. This disappointed me.’ (50 years, married, female)

## Discussion

Overall, the experiences of the women suggest that better measures should be taken to deal with side effects as in most cases, women stopped using Implanon because of side effects. Others use Implanon because it lasts longer and the majority of women are extremely satisfied with the use of Implanon. Most of the participants were satisfied with the assistance they receive at from healthcare facilities. It was found that most women heard about Implanon from their peers and from clinics. Amongst the issues that they had the common side effects were: menstrual bleeding, weight gain, and loss of sex drive.

The results reveal that most women were commonly having side effects whilst they were using Implanon contraceptive device. The side effects experienced varied for each individual. Amongst these side effects those commonly reported were vaginal bleeding/period disturbances, loss of body weight, headache and pain, memory loss or disturbances, insomnia and loss of sex drive. One of the widely reported side effect was menstrual bleeding or periods in most cases. The study participants reported that whenever they reach the clinic with menstrual bleeding or period disturbance, they were given contraceptive pills, which were helping them temporarily with the symptom, but after the completion of treatment the bleeding restarted. A descriptive study was conducted on healthcare provider communicator style and patient comprehension of oral contraceptive use. It was reported that there was poor instruction from healthcare practitioners with use of oral contraceptive pill that contributed to lack of understanding in patients. As a result, patients had poor recall or lack of motivation further resulting in failure (Schrader & Schrader 2001). However, the study’s findings also revealed that there was no improvement in the subsequent bleeding patterns. This shows that menstrual bleeding is a common problem to all women who use the implant and is a significant challenge that can be modified through treatment. Again treatment works over a short period, therefore the problem is solved temporarily. Then, the need for long lasting treatment is required to tackle the side effects of Implanon within the 3-year period. Conversely, a qualitative study conducted in 2010 (Spies et al. 2010) found that women were concerned with potential side effects and problems stemming from using new contraceptives. However, they required more information about long-acting reversible contraceptives related to side effects, how they work, length of use and how they might affect their fertility.

The literature revealed that women were more concerned with side effects that were related to the use of contraceptives. At the initial phase when screening and counselling is provided women are not adequately educated about side effects as a result they end up discontinuing the implant before its duration lapses. This is linked with a finding presented by Patel in 2014, which revealed that within the first 3 months after insertion of the Implanon it is common to experience side effects but women with the issue of bleeding patterns and other adverse effects were discontinuing the implant as they were not adequately counselled.

Other side effects reported were not so major in a way that could lead these women to remove the implant before its duration. The findings show that women were also experiencing other side effects such as weight loss, memory loss, loss of sex drive, headache and pains and insomnia. However, these side effects were not common with all participants hence they can be controlled and managed by adjustments with lifestyle. Therefore, the intervention requires properly taking history to identify other related causes that contribute to experiencing them. Similarly, a study conducted on women’s experiences with Implanon in South Nigeria revealed that most prevalent side effects experienced were vaginal spotting 60%, menorrhagia 13.3% and inter-menstrual bleeding 13.3% (Spies et al. 2010). However, there were other side effects reported but the bleeding disturbances contributed to 51.8% participants to change from Implanon to other contraceptives. Subsequently, there are various factors that are contraindicated to Implanon contraceptive, such as epilepsy, tuberculosis and antiretroviral drugs, poor screening and other lifestyle factors, such as substance abuse and alcohol consumption (Organon Laboratories Limited 2009). Therefore, the women may have not been sufficiently informed of other causes of pregnancy initially when they were introduced to Implanon.

Patni et al. (2006) found that Implanon’s® failure was because of potent enzyme inducers known to have deleterious effects resulting in intrauterine or ectopic pregnancy. The effects of pregnancy were directly related to drug interference of antiepileptic drugs (phenytoin, phenobarbital), antibiotics (rifampicin), antifungal drugs, protease inhibitors (efavirenz, nevirapine). Woolrych and Hill (2005) conducted a study to identify common reason for unintended pregnancy on Implanon users. The study’s findings showed 218 cases of unintended pregnancy, where 45 had insufficient data to assess the reason for contraceptives failure and 46 women were determined to have been already pregnant before Implanon insertion. There were 127 cases that were remaining and in some cases there was a failure to insert the implant in 84 women (Bradley, Croft & Rustein 2011). The other 19 cases were linked to incorrect timing of insertion, three cases were because of expulsion of Implanon and there were eight cases of interaction with hepatic enzyme-inducing medicines. Thus, this evidence shows that majority of the women may patiently continue to use the implant if they experience no changes with bleeding patterns. Other side effects are therefore regarded as modifiable. Conversely, most side effects were found to be experienced continuously without disappearing, although clients are informed that it is common within the first 3 months. Effective counselling and screening may improve contraceptive continuation even though there may be side effects that client’s experience (Landry, Wei & Frost 2008).

The study’s findings show that some participants were treated violently or harshly by their husbands at home when they discovered that their wives were using this implant. It was reported that one participant was forced to remove the implant because her husband told her that he would not continue paying *lobola* at home until she bears a child for him. Despite the situation she had at home, the nurses at the clinic deferred her booking date for the implant removal, making her anxious and concerned. Another situation was when a participant reported that her husband tried to remove her implant with a razor and needle. This clearly revealed that women experienced potential domestic violence and lack of support at home. In the first analysis it was evidenced that 69.1% of women stated that they had informed their partners or husbands about their decision to use the implant, whilst 30.9% also responded that their partners were not aware that they were using it. Peel and Moreje (2013) argued that South African society, particularly in rural areas, is still male-dominated, and that women feel forced to prove their fertility. Kamal and Lim (2010) revealed that a husband’s approval of family planning is taken as a pivotal determinant of women’s contraceptive use.

Williams et al. (2008) reported that women experience limited autonomy, mobility and power related to household and fertility decision-making. The autonomy described by women is subversive and nested within a framework where they are not able to make overt decisions about their bodies, often manifested by covert contraceptives. According to one of the participant, some husbands feel that a wife should always listen to them and obey them. Kamal and Kim (2006) argued that men dominate decision-making regarding family size and their partner’s use of contraceptive methods, amongst a variety of traditions. It was found that men attempt to prevent women from using contraceptives. It was also evidenced that certain men even fight against women in trying to prevent them from visiting the clinic for family planning. In some instances, some men destroy clinic cards or contraceptives received from the clinic because they want to prove their fertility as the result of their actions (Williamson et al. 2009).

## Limitations

The study was conducted in one community healthcare clinic found in Pietermaritzburg in KwaZulu-Natal. Therefore, the research findings cannot be generalised to all community healthcare clinics in this province. Furthermore, the small sample size was also a possible limitation.

## Implications for future research

A challenge for the future research must be on implementation of effective screening and training or retraining programme of all healthcare professionals especially in primary healthcare clinics to prevent high numbers of clients discontinuing Implanon contraceptive device. Furthermore, there is a great need to evaluate contraceptive policy and guidelines with a view to come up with long-acting contraceptive method in the health system. The study’s findings can be used by other researchers, health professionals, government and global authorities.

## Recommendations

These recommendations are proposed as interventions that will assist to improve standards in the service provision of Implanon contraceptive device. Therefore, these interventions were made based on the study’s findings in this research investigation:

- Effective screening and counselling still need to be strengthened strongly to prevent adverse outcome with Implanon use amongst women. This investigation found that most of the issues arising with Implanon could have been prevented initially if proper screening and counselling were performed such as: proper history taking and performing investigations required in screening and educating. In addition, women should be counselled about the side effects to be expected initially and should be informed about possible treatments to correct the side effects.
- All healthcare practitioners and staff should always support those women coming to clinic subsequent to side effects. Staff must show empathy and should be non-judgemental with a professional attitude towards any client. It is the role of a nurse to listen attentively and communicate friendly with clients and suggest strategic ways to solve all issues raised by the clients. No clients should leave the health facility without receiving sufficient assistance.
- The national health government should seek strategies that will stop uncontrollable side effects such as menstrual bleeding in clients. There should be a hierarchy when dealing with bleeding issues where the first line of option should be a general treatment and second line should be intense treatment thereafter implant should be removed especially those with constant heavy bleeding that does not subside. Problems could arise if clients could end up staying at home whilst bleeding profusely as they may be avoiding healthcare practitioners because of their attitude.
- The need for spousal support should be explored broadly therefore clients must be encouraged to bring their partners when coming for contraceptives to create the awareness and support from spouse. Women should be advised to come with their partners when they have to decide on contraceptive method they want.
- Booking for removal of implant should not be scheduled very far in cases where clients are having negative experiences with the implant. At least clients should be free to remove the implant any day they want without any pressure to continue to use the implant.
- All healthcare providers especially nurses working in primary healthcare facility should receive in-service training on the use of Implanon. This can help to achieve the one-stop shop strategy whereby the client should receive all relevant help, they came for in the clinic in one consultation room and from one healthcare provider. This will also allow the health staff to work as a team when dealing with various conditions and in solving side effects or issues brought forward by clients.
- Effective screening tools should be in place and developed specifically for Implanon to allow health care practitioners to easily adopt and understand the entire implant process.
- Body weight of all clients who used the implant should be strictly monitored, even in subsequent visits when they come with side effects. Healthcare workers should constantly monitor their weight.
- It is essential at the initial phase before Implanon insertion that the clients are asked to specify all medication they are taking and further educate the clients as to which medications are contra-indicated with Implanon contraceptive method.
- Awareness campaigns should also be organised by coordinators to increase awareness and knowledge about the implant. School health teams, community health workers, nurses and other members should work to achieve awareness in library, schools, community and church.
- Imperative research investigation should be conducted broadly to explore Implanon contraceptive method involving more health facilities and clients. Investigation should involve healthcare providers as to issues and experiences they might have regarding this new contraceptive method.

## Conclusion

The National Department of Health must introduce a proper and effective screening tool and reinforce staff training to improve the uptake of Implanon. There is a need to revise guidelines in management of side effects of Implanon as a major intervention. Effective measures should be taken to strengthen partner support and awareness in the society.
